# Uremia-Associated Immunological Aging and Severity of COVID-19 Infection

**DOI:** 10.3389/fmed.2021.675573

**Published:** 2021-04-14

**Authors:** Michiel G. H. Betjes

**Affiliations:** Division of Nephrology and Transplantation, Department of Internal Medicine, Erasmus Medical Centre, Rotterdam, Netherlands

**Keywords:** uremia, chronic kidney disease, thymus, adaptive immunity, lymphopenia, immunological aging, COVID-19

## Abstract

One year after the start of the COVID-19 pandemic it has become clear that some groups of individuals are at particular high risk of a complicated course of infection resulting in high morbidity and mortality. Two specific risk factors are most prominent, old age and the presence of co-morbidity. Recent studies have shown that patients with compromised renal function, especially those treated with renal replacement therapy or having received a kidney transplant are at a much higher risk for severe COVID infection and increased mortality. This may be in part due to the increased prevalence of co-morbid conditions in these patients but specific alterations in their immune system, reflecting premature immunological aging, may be equally important. In this review the different aspects, in particular thymus function and memory T cell expansion, of uremia-associated immunological aging are reviewed with respect to COVID 19 infection. In essence, the decreased generation of naïve T cells may be instrumental in suboptimal anti-viral immune responses while the relatively uncontrolled expansion of effector T cells may facilitate the feared phase of the COVID-19 infection with excessive and live-threatening inflammation of the lung parenchyma.

## Uremia-Associated Immunological Aging

### General Aspects

End-stage renal disease is associated with increased risks for infections, cancer and a poor vaccination response to vaccines like Hepatitis B surface antigen (HBsAg) (1). The accumulation of uremic toxins and increased oxidative stress leads to a pro-inflammatory state which is believed to underlie the impaired immune system. Uremia affects all aspects of both the innate and adaptive immune system [reviewed in (1)]. Cell numbers of innate immune cells like monocytes and granulocytes are normal to increased. However, these cells have a more activated profile with expansion of, for example, the subset of pro-inflammatory monocytes CD14posCD16pos while their functionality may be comprised (2, 3). Dendritic cells are professional antigen presenting cells and at the crossroad of the innate and adaptive immune response. In particular the subset of lymphoid dendritic cells is affected by aging and uremia as opposed to the myeloid dendritic cells (4–7). These lymphoid dendritic cells produce large amounts of type 1 interferon and are key for adequate antiviral responses (8). In addition, there are less dendritic cells present in the skin and circulation which may contribute to a less efficient adaptive immune response (5, 9).

Progressive lymphopenia with relatively more highly differentiated memory T cells is observed in association with more advanced stages of chronic kidney failure (10–12). Tracking the anti-HBsAg T cells after vaccination in patients with renal failure showed an insufficient CD4 T cell response which correlated closely with an impaired serological response (13).

**Figure 1:**
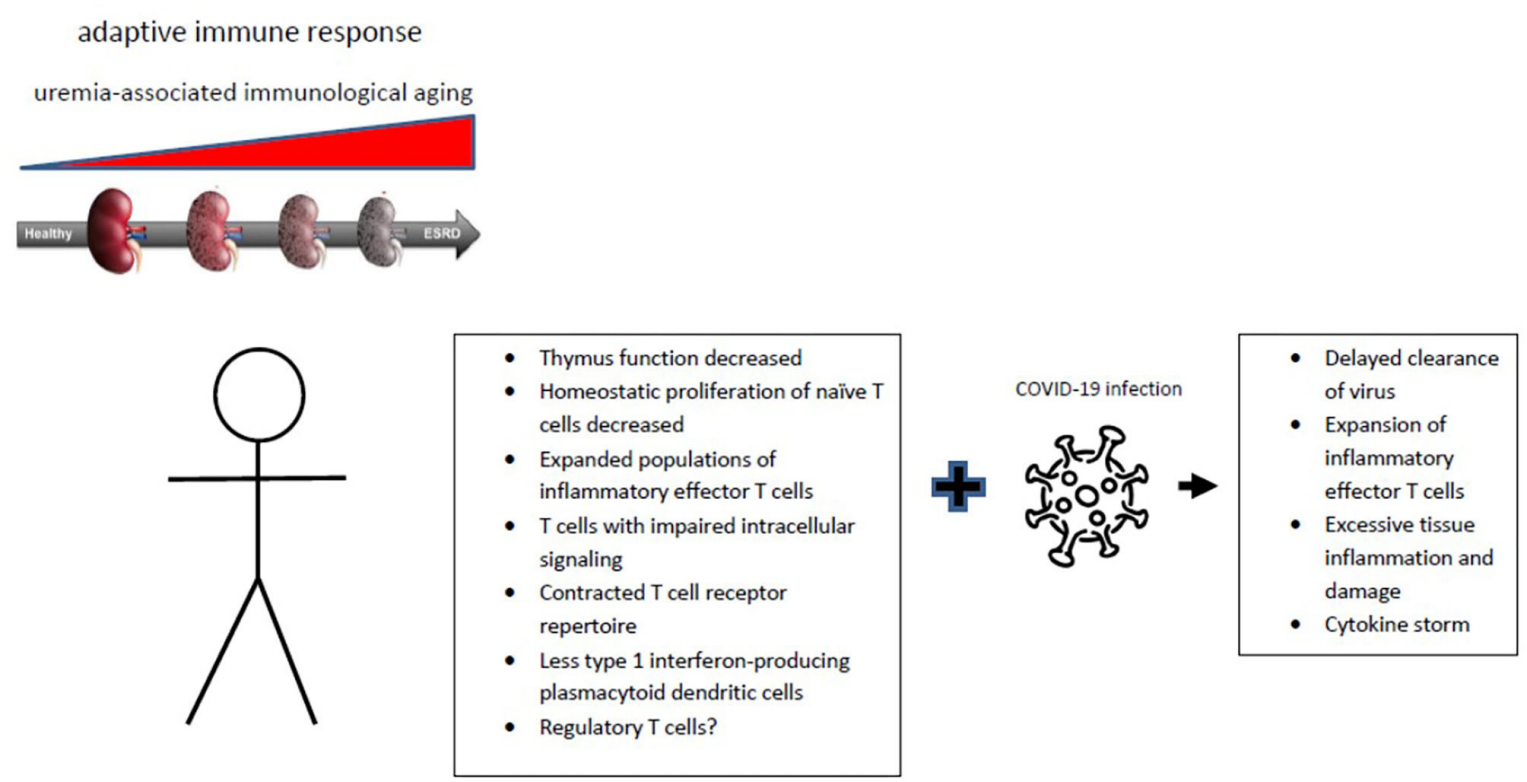
Progression of chronic renal failure is associated with immunological aging affecting the adaptive immune system. The typical hall marks of immunological aging are given with an uncertain role for regulatory T cells. The changes of uremia-associated immunological aging can contribute to the substantially increased COVID-19 infection-associated morbidity and mortality of patients with chronic renal failure.

The changes within the adaptive immune system and consequences for immune responses closely resembles the effects of aging (Figure 1) (14). A shift in favor of myeloid vs. lymphoid precursor hematological stem cells in the bone marrow may be important (15). This process is driven by epigenetics which in turn is under the influence of systemic inflammation and oxidative stress as observed in end-stage renal failure (16, 17). However, the adaptive immune system is more broadly affected by aging with thymus involution as a major cause of a decreasing output of naïve T cells, in combination with increasing numbers of memory T cells and changes in the regulatory T cell compartment. The first two observations are consistently found in the elderly and patients with end-stage renal failure. The expansion of memory T cells in elderly individuals is usually associated with a slight increases in markers of systemic inflammation and therefore frequently named inflamm-aging (18).

The T cell system is studied the most intense in aging research as peripheral blood is an easily accessible source of abundant T cells and many assays are available to study phenotype, differentiation status and function of T cells. In fact, such an integrative analysis of the T cell system of ESRD patients showed immunological aging by an average of 15–20 years, meaning that the composition of the population of circulating T cells of a 50-year old hemodialysis patient resembles that of a 70-year old healthy individual (11, 19). Of note, the inter-individual variation is substantial and for instance individuals with a genetic background of longevity show less signs of immunological aging (20).

### Thymus Function and Aging

The thymus is important in producing naïve T cells which all have a specific T cell receptor (21). Naïve T cells leaving the thymus are called recent thymus emigrants (RTE) and were positively selected for the capacity to interact with the HLA molecules of the antigen-presenting cells but deleted if this interaction was to strong, thereby preventing potential dangerous autoreactivity. In addition, regulatory T cells (Tregs) are also generated which are called natural Tregs (22). Essentially, the thymus continuously generates the enormous diversity of T cell receptors which is needed to combat efficiently the wide variety of pathogens that may be encountered while controlling autoreactive T cells (23).

Aging is invariably associated with involution of the thymus leading to a steady decline in RTEs. The RTEs can be detected in the circulation by, for example, expression of CD31 on naïve T cells and there is on average an almost linear relationship between decreasing numbers of CD31 positive naïve T cells and age (24, 25). Homeostatic proliferation, particularly of CD4 naïve T cells, is able to maintain the volume of the naïve T cell compartment but naïve CD8 T cells become severely depleted in the elderly (26–29). This may lead to a contraction of the TCR repertoire which in turn can limit the diversity and thereby the efficacy of the immunological response (30). For instance, thymus output is crucial in the formation of protective immune responses during the early formation of a *Salmonella* infection but is dispensable once persistent *Salmonella* infection is established (31). Whether the output of natural Tregs is in line with the decrease in RTE's is not known.

Progressive severity of renal failure is associated with decreased numbers of naïve T cells which can be attributed to both a decrease in RTE's and a decrease in compensatory homeostatic proliferation (11, 32, 33). Activation-induced cell death of naïve T cells is increased and likely underlies part of the pathogenesis (11). As a result, at all decades of life end-stage renal failure results in a significant contraction of the circulating naïve T cell compartment (33, 34). Recent studies have shown that lymphoid and non-lymphoid tissues may also harbor naïve T cells and the relation with circulating naïve T cells is not clearly established (28, 35). However, comparing the percentage of lymph node naïve T cells with the percentage of peripheral blood naïve T cells showed a very close correlation (36).

Why the production of RTE's is affected by uremia is not known but from animal experiments it is clear that loss of renal function leads to volume loss of lymphoid organs like the thymus (37). The thymus and in particular the thymus epithelial cells appear to be very sensitive for inflammatory and oxidative stress which translates into an increased tendency for apoptosis (38–43). This may not only explain the thymus involution with normal aging, as a result of prolonged and variable exposure to these conditions, but also the uremia-associated decrease in thymus function in parallel with the increased pro-inflammatory environment observed with progressive loss of renal function.

Naïve T cell numbers in both healthy individuals as in recipients of kidney transplants were independently associated with all-cause mortality (19, 33, 44). Although this implies a causal relationship, this is not without uncertainty. Life events leading to episodes of increased inflammation and oxidative stress could accelerated thymus involution leading to “low for age” numbers of naïve T cells but also have a broad negative impact on the physical robustness of an individual. Therefore, the possibility exists that low naïve T cell numbers not only contribute to a weakened immune system but also point to a life history with harmful events leading to frailty (45–47).

### Memory T Cell Expansion and Immunological Aging

The second hall mark of an aged T cell system is the expansion of memory T cells which may show signs of senescence and/or exhaustion which can be defined as the loss of proliferative capacity and specific effector functions like cytokine production and cytotoxicity (48). The increased numbers of memory T cells arise during life as a natural consequence of an immune system that has reacted to specific pathogens. When infections persist, like chronic hepatitis C or HIV, it may lead to progressive differentiation of virus-specific memory T cells into senescence and susceptibility for cell death (49–51). With increasing age, the memory T cells have undergone many rounds of replication with consequent shortening of their telomeres (52). Measuring telomere length therefore provides another measure of immunological aging (11).

Expansion of particular populations of memory T cells in the elderly persons may lead to a skewed TcR repertoire and may cause gaps in the TcR repertoire (53–56). The latter could fit in the concept of immunological space, which postulates that the immune system can only support the survival of a certain quantity of immune cells (57, 58). Of note, in recent years it has become evident that all tissues harbor a large quantity of resident T cells that do not circulate and that provide local protection against pathogens (59). As a first line of defense, the resident T cells are enriched in antigen-specific T cells that react to pathogens which are frequently encountered within that tissue, for example influenza-specific T cells in the lungs (60, 61). In contrast, relatively few highly differentiated T cells are present in the lymph nodes (36). Thus, the population of circulating T cells is only one of the many compartments of T cells, but easy to monitor and in general reflecting an ongoing immune response by increased frequencies of antigen-specific T cells.

Several studies have shown that an expanded population of differentiated effector memory T cells which have lost the expression of the co-stimulatory molecule CD28 is associated with less efficient vaccination and a decreased risk for rejection after kidney transplantation (14, 62–65). The underlying mechanisms may be multiple as the pool of CD28null T cells harbors many different cell types including senescent T cell and cells with a regulatory function (66, 67).

Circulating numbers of natural Tregs may increase with age as a result of an expanded population of memory Tregs. These inflated numbers of Tregs in the elderly can limit immune responses like vaccination response to influenza but could also foster autoimmunity and chronic inflammation (68). Chronic renal failure *per se* does not affect numbers and function of circulating natural Tregs (69).

Of interest is the observation from animal experiments and young adults after thymectomy at childhood, that lower numbers of naïve T cells facilitate an expansion of circulating memory T cells which may be a relevant phenomenon in immunological aging (70–72).

In patients with chronic renal failure the immunological aging of the memory T cells is more advanced as can be shown by the increased reduction in T cell telomere length and a higher frequency of highly differentiated T cells (2, 11, 19, 73). In addition, as in healthy individuals, the important intracellular signal pathway involving the MAP kinases ERK, p38 and DUSP6 is unfavorably changed by aging (74, 75).

## Implications for COVID-19 Infection

Increasing morbidity and mortality associated with COVID-19 infection is highly associated with elderly age and co-morbid conditions (76). Patients on dialysis, with CKD and recipients of organ transplant represented three of the four comorbidities associated with the highest mortality risk from COVID-19 (77). Most likely, this is at least in part associated with their prematurely aged immune system as a coordinated adaptive T cell response is associated with less severity of disease (78). Of note, changes at many levels of the immune system other than the adaptive T cell response have been described in association with aging and could contribute to severity of COVID-19 infection, but their relative importance has as yet not been established.

Fatality of COVID-19 infection is highly associated with a dysregulated immune response with progressive and severe inflammation of the lung parenchyma leading to extreme hypoxia (79–81). Several mutually not exclusive scenarios may lead to this outcome in the context of immunological aging. First, the aged immune system may be slow or inefficiently responding to this new viral pathogen as a result of a contracted TcR repertoire in the naïve T cell population and a general decline in T cell function by less effective intracellular signaling. In addition, the decreased numbers of plasmocytoid dendritic cells may have a profound negative effect on viral control as type 1 interferon is important in COVID-19 clearance in an experimental hamster model of infection (82).

Both deficiencies would lead to delayed clearance of the virus and prolonged stimulation and expansion of memory T cells that are COVID-19 reactive. On average lower numbers of T cells have been found in hospital-admitted COVID-19 patients and lower cell numbers, specifically naïve T cells, are related to disease severity (78, 83–88). Although of considerable interest, these observations are most likely caused by the COVID-19 infection itself which leads to decreased T cell numbers which may restore with clinical improvement (85, 89).

Second, immunological aging can lead to a T cell system prone to expansion of highly reactive memory T cells as regulation by Tregs is less efficient, low numbers of naïve T cells facilitate such a response and time to resolution of the viral infection is relatively slow. This scenario is not just hypothetical as shown by the large number of studies on infection with cytomegalovirus. Infection with this herpes virus typically leads to a strong T cell immune response which can be recognized by an expansion in the peripheral blood of both highly differentiated memory CD8 and CD4 T cells (90, 91). The CMV reactive CD4 T cells can be readily detected as they are negative for the co-stimulatory molecule CD28 (CD4posCD28null T cells)(90). Both the infectious dose and the age of the individual are related to the expansion of CMV-specific memory T cells (92, 93). Specifically, in elderly patients with chronic renal failure the expansion of CD4posCD28null T cells which normally comprise <1% of the CD4 T cell population may be such that over 50% of CD4 T cells become CD28null (94, 95). The CD4posCD28null T cells are highly cytotoxic and express the chemokine receptor CXCR3 which allows for migration over endothelial cells (96). These cells are not without harm as they have been identified as a non-classical risk factor for atherosclerotic disease probably by their capacity to destabilize atherosclerotic plaques (97, 98). Thus, CMV infection in immunologically aged individuals like patients with chronic renal failure may cause poorly controlled memory T cell expansion with subsequent collateral damage in patients with atherosclerotic plaques.

Such an exaggerated and harmful T cell immune response in elderly COVID-19 patients with a severe course of disease is of course much more acute and intense leading to expansion of highly activated memory T cells in association with a cytokine storm (88). In the case of COVID-19 the large expansion of highly reactive effector T cells is likely primarily present in the lung parenchyma as has, for example, been shown for influenza-specific T cells. Therefore, peripheral blood COVID-19 antigen-specific T cells are a reflection of the intensity of the immune response which may show different characteristics and may be much worse at the tissue level (99–102).

In the case of severe COVID-19 infection, controlling the inflammatory response by high dose steroid is currently the best option (103). A recent study among recipients of a liver transplant with COVID-19 infection showed that the use of tacrolimus was associated with a significant reduction of mortality (104). This findings underlines that limiting the excessive T cell response, in this case by tacrolimus, is a key element in harnessing the morbidity and mortality of COVID-19.

## Therapeutic Strategies to Influence Uremia-Associated Immunological Aging

Reversing immunological aging in humans is currently not possible although some interventions may be beneficial (40, 105). As thymus involution underlies ever decreasing naïve T cell numbers with aging and possible contributes to memory T cell expansion it would be of prime importance to control this process. The biological process of thymus involution is now better understood and it is clear that loss of thymus epithelial cells is essential.

Recent studies have shown that thymus involution involves the aging of the stromal microenvironment formed by thymus epithelial cells (TEC)(105). Many factors like cytokines, sex steroids and transcription factors are likely involved in TEC aging (106). Expression of the TEC autonomous transcription factor FOXN1 is pivotal for differentiation and maintaining TEC integrity. A null mutation of FOXN1 in mice results in a lack of hair and thymus, and gradual excision of FOXN1 over time in an experimental model results in thymus involution (107, 108).

This process can be favorably attenuated by transfecting thymus cells with FOXN1(70) and cellular therapy with FOXN1 producing stem cells or cytokine-to-TEC-based therapies using IL-22 or keratinocyte growth factor have shown promising results in experimental models. These approaches offer at least proof of the concept that thymus function can be (partially) restored (106).

Interleukin 7 is an important cytokine for T cell proliferation and homeostasis. Administration of recombinant IL-7 in humans appears to be safe and increases peripheral T cell numbers. However, there is little direct impact on thymus function which limits its use as a regenerative cytokine for the involuted thymus (109). Of interest, targeting of IL-7 to the thymus, for example, by a plasmid-delivered IL-7 fusion protein, was able to restore the thymus architecture and cellularity in aged animals (110).

Restoring renal function by kidney transplantation leads to a rapid clearance of inflammatory cytokines and relieves oxidative stress in ESRD patients. However, there is no reversal in any of the markers of T cell aging even at 1 year after transplantation (111). Thus, once established, thymus involution seems irreversible, leaving the ESRD patient with premature aging at a persistent increased risk for mortality, even after regaining adequate renal function with a GFR over 60 mL/min. The underlying mechanisms are likely epigenetic changes induced by any combination of inflammation and oxidative stress associated with uremia, which are not easily reversible (1).

Of considerable interest is a recent observation that a healthy lifestyle may slow down thymus involution. Smoking and obesity are associated with fattening of the thymus (112) and bariatric surgery can partly reverse immunological aging (113). An observational study showed that elderly individuals with a high intensity of daily exercise had a better preservation of thymus function and less senescence of their immune system (114, 115). Having a healthy lifestyle with sufficient exercise will likely not reverse an atrophied thymus in ESRD patients but may delay further involution. Differences in lifestyle may also be part of the explanation for the substantial inter-individual variation observed at every decade of life in the number of naïve T cells and RTE's.

## Conclusion

Aging of the T cell system has specific hall marks and is largely characterized by a progressive decrease of thymus function and expansion of highly differentiated memory T cells. Patients with renal failure, even after successful kidney transplantation may have severe premature immunological aging in particular in association with CMV infection. Immunological aging may explain why severity of COVID-19 infection is both age dependent and significantly increased in patients with chronic renal failure.

## Author Contributions

The author confirms being the sole contributor of this work and has approved it for publication.

## Conflict of Interest

The author declares that the research was conducted in the absence of any commercial or financial relationships that could be construed as a potential conflict of interest.
